# Biochemical reconstitution of temozolomide-induced mutational processes

**DOI:** 10.1016/j.jbc.2025.110676

**Published:** 2025-09-04

**Authors:** Mahima R. Sanyal, Tomohiko Sugiyama

**Affiliations:** 1 Department of Biological Sciences, Ohio University, Athens, Ohio, USA; 2 Molecular and Cellular Biology Graduate Program, Ohio University, Athens, Ohio, USA

**Keywords:** *in vitro* mutagenesis, temozolomide, cancer mutational signature, O^6^-methylguanine, methyl guanine methyltransferase, DNA methylation, DNA polymerase, DNA repair

## Abstract

Temozolomide (TMZ), a DNA alkylator, is a chemotherapeutic agent for brain tumors, but the treatment induces a distinct pattern of mutations, known as a cancer mutational signature single-base substitution signature 11 (SBS11). Although the correlation between TMZ treatment and SBS11 mutations is very clear, the precise biochemical mechanisms that cause SBS11 have not been elucidated. TMZ can alkylate DNA at several locations, among which O^6^-methylguanine is believed to be the most toxic. In this study, we reconstituted potential biochemical processes of TMZ-induced mutagenesis *in vitro*, including TMZ-induced DNA damage and subsequent DNA synthesis by various DNA polymerases. Next-generation sequencing of the DNA products revealed mutations with a similar spectrum to SBS11. Efficient production of the SBS11-like mutation spectra required DNA in double-stranded form and multiple exposures to TMZ. Replicative polymerase δ alone generated SBS11-like mutations on the damaged DNA. Most SBS11-like mutations were sensitive to methyl guanine methyltransferase treatment, indicating that the mutations are formed on O^6^-methylguanine modifications. Human Pol η reduced the SBS11-like mutations, indicating its suppressive role in TMZ-induced mutagenesis. Yeast Pol ζ and human Pol κ generated distinct mutations unrelated to SBS11.

Temozolomide (TMZ) is a widely utilized adjuvant chemotherapeutic agent effective in the treatment of central nervous system tumors, metastatic gliomas, refractory leukemia (1), and metastatic melanoma (2, 3, 4). Its efficacy for treating brain tumors is largely attributed to its ability to penetrate the blood–brain barrier, resulting in nearly 100% bioavailability in cerebrospinal fluid (5). TMZ adds methyl groups onto DNA bases, and its mode of action involves its spontaneous hydrolysis (half-life ∼1.8 h) into methyl-triazenylimidazole-carboxamide, which is further decomposed into 4-amino-5-imidazole-carboxamide and a methyldiazonium cation, the latter having a half-life of 2 min (6) and being the active methylating agent. The therapeutic effects of TMZ are derived from DNA methylation that eventually induces apoptosis and senescence in cancer cells. Recent pharmacokinetic models suggest that repeated low-dose administration of TMZ may optimize therapeutic outcomes, specifically in the treatment of malignant gliomas (5). However, it is associated with significant drawbacks, including high rates of tumor relapse and toxicity (6). These findings align with clinical observations of TMZ usage in therapeutic settings, where its benefits are often counteracted by challenges, such as resistance development, recurrence of malignancies, and treatment-associated toxicity (7, 8).

Direct biochemical studies of TMZ-induced DNA damage are limited; much of the current understanding has been deduced from studies on the general action of other SN1 methylators, which have similar reaction modes. They generate a variety of adducts, including N7-methyl guanine (N7-meG), which is the predominant adduct, followed by N3-methyl adenine and O6-methyl guanine (O6-meG), as well as other minor adducts (N1-meA, N7-meA, N1-meG, N3-meG, N3-meC, O^2^-meC, N3-meT, O^2^-meT, and O^4^-T) (9, 10, 11). To quantify the extent of DNA methylation damage and to differentiate the activity of methylators, studies often rely on the O6-meG/N7-meG ratio. This metric distinguishes SN1 and SN2 methylators, with reported values of 0.11 for SN1 methylators like TMZ and 0.004 for SN2 methylators (12). The higher O6-meG/N7-meG ratio associated with SN1 methylators reflects their characteristic propensity to generate O^6^-meG lesions, which are critical to their cytotoxic effects. The proportions of adducts formed by SN1 methylators also vary depending on tissue type and experimental system. For example, the proportion of N3-methyl adenine as a result of SN1 methylation varies from 9% in cell-free systems to 4% in cultured cells and 3% in rat liver DNA (12). Similarly, O^6^-meG adduct constitutes 5% to 7% of total adducts produced in cell-free systems after TMZ treatment. *In vivo*, O^6^-methylguanine (O^6^me-G) lesions also vary across different tissues like the liver, lung, and kidneys (13). These variations in adduct formation highlight the complexity of TMZ-induced DNA damage and underscore the need for further direct biochemical studies.

The major repair mechanism of O^6^-meG lesions involves O^6^-methyl guanine-DNA methyltransferase (MGMT), which removes the O^6^-methyl group from O^6^-meG lesions, thereby restoring guanine to its original structure (14, 15, 16). When MGMT expression is suppressed, O^6^-meG lesions can persist, leading to the erroneous incorporation of dTMP opposite the lesion. The mispaired base can be removed by the mismatch repair (MMR) system, leaving the lesion intact. This leads to a futile cycle of misincorporation and correction that triggers a cytotoxic response to TMZ in cancer cells (17, 18, 19). Interestingly, cells deficient in MMR can tolerate O^6^-meG lesions, allowing DNA adducts to persist without triggering significant cytotoxicity (20, 21, 22). In contrast, functional MMR machinery exacerbates the cytotoxic effects of O^6^-meG lesions by inducing chromosomal aberrations, including DNA strand breaks and sister chromatid exchanges (20).

In hypermutated tumor samples, TMZ has been associated with C>T (G>A) mutations (23). More extensive analyses of cancer genomes revealed mutational signatures, which provided a better understanding of the mutational processes occurring in cancer (24). Single-base substitution signature 11 (SBS11) has been linked to TMZ-treated cancer samples (24, 25). The vast majority (99%) of TMZ-treated diffuse gliomas showed hypermutation (26, 27), and nearly 97% of mutations in hypermutated recurrent cancers were due to SBS11-like mutational patterns with increased C>T transitions at CpC and CpT sites (28). These studies indicate a causal relationship between TMZ and SBS11. Unexpectedly, exposure of TMZ to induced pluripotent stem cells did not reproduce an SBS11-like mutation spectrum (29).

While it is known that O^6^-meG is the major adduct that causes cytotoxicity, it is less clear which TMZ-induced adduct is directly involved in SBS11. In addition, the enzymatic mechanisms that convert TMZ-induced DNA damage into mutations have not been clearly demonstrated (30, 31). Especially, the DNA polymerases that are directly involved in the production of SBS11 mutations have not been identified. Here, we employed a cell-free system to reproduce TMZ-induced mutations. While the *in vitro* system did not include many cellular mechanisms that potentially modulate mutagenic processes, we believe that it demonstrates potential core reactions of TMZ-mediated mutagenesis.

## Results

### Replicative DNA polymerase can produce mutations on TMZ-damaged DNA *in vitro*

**Figure 1:**
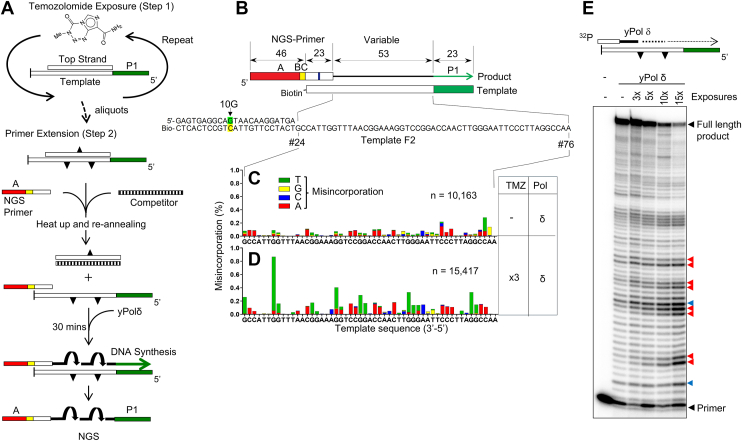
**Experimental design and examples of TMZ-induced DNA damage and mutagenesis *in vitro*.***A,* illustration of repeated TMZ exposure on DNA templates (step 1) and primer extension (step 2). *B,* structure of a synthetic DNA substrate (template F2) and an NGS primer. The NGS primer contained an A-adaptor (*red box*) and a barcode sequence (*yellow box*). The template contains the P1 adaptor (*green box*) for NGS. The 3′-end of the template was modified with biotin to minimize template extension. The sequences of other templates are shown in Fig. S1. *C* and *D,* single nucleotide substitution frequencies were plotted at each base of the 53-nt variable region (position #24–76 of *B*) of the template F2. TMZ-exposure conditions and polymerase used for primer extension are indicated on the *right* of the graph. *E,* after multiple exposures with TMZ, dsDNA (template F2) was subjected to the primer extension from the ^32^P-labeled primer by yPol δ. NGS, next-generation sequencing; TMZ, temozolomide; yPol δ, yeast Pol δ.

**Figure 2:**
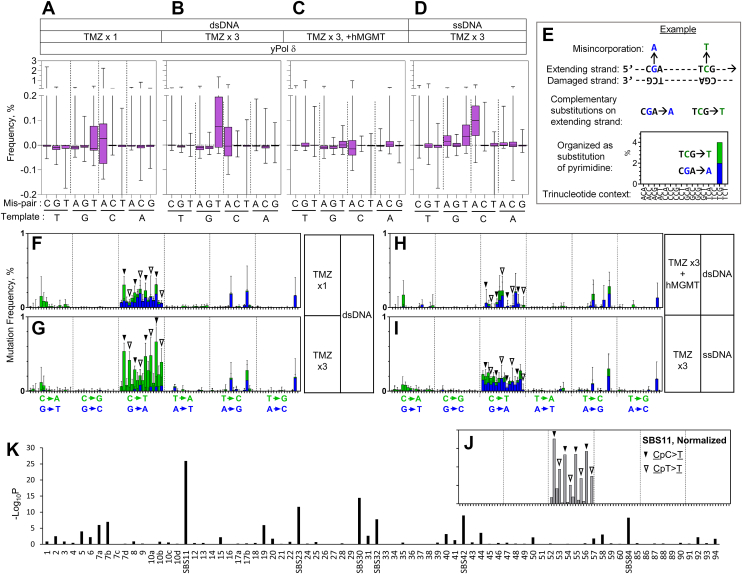
**TMZ-induced mutation spectra.***A*–*D,* frequencies of mutations that were produced by yPol δ on dsDNA (*A*–*C*) or on ssDNA (*D*) that were exposed to TMZ once (*A*) or three times (*B*–*D*) and followed by hMGMT treatment (*C*), were shown by box and whisker plots. Boxes are first-third quartiles, whiskers are highest and lowest, and lines in the boxes are medians. Numbers of A, C, G, and T residues of the templates are 88, 90, 88, and 84, respectively. *E,* an example demonstrating how the *in vitro* mutation spectrum was obtained. In this example, strand extension occurs from *left* to *right* on the damaged template. During TLS, two misincorporations (G>A in *blue* and C>T in *green*) occur on the extending strand. These changes are complementary to each other, including the sequence context, making them indistinguishable by genome sequencing. Since our *in vitro* approach separately quantifies such complementary mutations on the extending strand, we show them at the same position on the graph as a *blue bar* (purine substitution) and a *green bar* (pyrimidine substitution). *F*–*I*, the single-base substitution frequencies in *A*–*D* are reorganized with trinucleotide contexts as explained in *E*. *Filled and open triangles* are NpCpC>T and NpCpT>T mutations, respectively. *J,* COSMIC cancer signature SBS11 (version 3.2) after normalization of the appearance of trinucleotide sequences in the human genome. *K,* similarities between *G* and cancer signatures. Signatures with *p* > 10 are labeled. hMGMT, human methylguanine methyltransferase; TLS, translesion synthesis; TMZ, temozolomide; SBS11, single-base substitution signature 11; yPol δ, yeast Pol δ.

**Figure 3:**
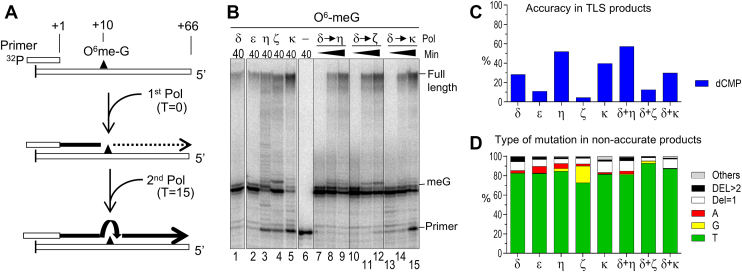
**Primer extension and NGS analysis of O^6^me-G at a defined position.***A,* illustration of the system. The exact structure of the template is shown in Fig. S1*B*. *B,* primer-extension reactions from the ^32^P-labeled primer. Lanes 1 to 5 show the single polymerase reactions in which the indicated polymerases were incubated with the primer–template complex for 40 min. Lanes 7 to 15 showed two polymerase reactions in which indicated polymerases were used as first and second polymerases, and reactions were stopped at T = 10, 20, and 40 min. Note that T = 10 min sample did not include the second Pol, which was added at T = 15 min. *C,* the same template used in *B* was hybridized with NGS primers and extended by the indicated polymerases, and frequencies of dCMP insertion at O^6^-meG were calculated from NGS reads. *D,* percentages of mutation types in the DNA products that did not receive dCMP at the O^6^-meG were calculated from the same dataset shown in *C*. NGS, next-generation sequencing; O^6^me-G, O^6^-methylguanine.

To analyze TMZ-mediated DNA damage and mutagenesis *in vitro*, we used a cell-free system in which synthetic dsDNA substrates were exposed to TMZ. Because TMZ spontaneously breaks down (*t*_1/2_ = 1.8 h) to its active methylating compound that has a very short half-life (*t*_1/2_ = 2 min) (6), we exposed the DNA substrate to TMZ several times to increase the chance of DNA damage (Fig. 1*A*, step 1). For each exposure, 400 μM of TMZ was added to DNA and incubated for 1.5 h at 37 °C. For multiple exposures, the same treatment was repeated every 1.5 h. Then, the DNA was purified, and the extent of damage was estimated by primer extension assay using yeast Pol δ (yPol δ) (Fig. 1*E*). As the exposure increased, the amount of full-length DNA products decreased and shorter DNA increased. The most prominent sites that blocked yPol δ were G residues of the template (*red triangles* in Fig. 1*E*), confirming that TMZ mainly modifies G residues, whereas yPol δ extension was also blocked at some A residues (*blue triangles*). This assay provided insight into the extent of TMZ-induced damages, helping to optimize the conditions for the mutation assay. However, this is not an accurate quantification of TMZ-induced DNA damages, since yPol δ can bypass the damage at low efficiency (Figs. 2 and 3).

To analyze the mutations on the primer-extension products, both damaged and undamaged DNA were subjected to primer extension using next-generation sequencing (NGS) primers (Fig. 1, *A* and *B*). The resulting fully extended products were then analyzed with an Ion Torrent NGS system. Since the NGS primer contains an A adaptor and the template includes a P1 adaptor, only fully extended products are recognized by the NGS system (32, 33, 34, 35). In addition, the NGS primer for each reaction had a unique barcode so that the products of separate reactions were readily identified in pooled NGS reads. Figure 1, *C* and *D* shows examples of the results, showing the frequency of single-base substitutions that were introduced by yPol δ on the undamaged template (C) and the template exposed to 400 μM TMZ for 1.5 h three times (D). The mutations are mapped on the 53-nucleotide “variable” sequence of the template and expressed as a percent of substitution in fully extended products (Fig. 1*B*). As shown in Figure 1*D*, yPol δ misincorporated dTMPs (*green bars*) at G residues of the TMZ-treated template, causing C>T mutations.

### yPol δ produces an SBS11-like spectrum of mutations on TMZ-damaged templates

For more quantitative analyses of the mutation frequencies in diverse sequence contexts, TMZ-induced single-nucleotide substitution frequency data were collected from seven templates with different sequences at the 53-nt variable region (templates A2–G2; Fig. S1). At each base, background mutation frequencies on untreated templates were subtracted from those on TMZ-treated templates. The resulting TMZ-induced mutation frequencies across all bases in the variable regions of the seven templates (Fig. S2) are summarized in Figure 2, *A*–*D*. Since TMZ is a proposed etiology of SBS11, it is of special interest to examine the similarity between our *in vitro* mutations and cancer signatures. To do this, we reorganized the dataset in Figure 2, A–D into a comparable format to the cancer signatures, as described in our previous studies (32, 33, 34, 35) (Fig. 2*E*). One of the resulting *in vitro* mutation spectra (Fig. 2*G*) showed C>T mutations in the NpCpC and NpCpT contexts (indicated by *filled and open triangles*, respectively), which is characteristic of SBS11 (Fig. 2*J*). This SBS11-like mutation spectrum was produced by yPol δ-mediated primer extension on dsDNA templates that were exposed to TMZ three times. The cosine similarity of Figure 2*G* to SBS11 is 0.854 (-log_10_*p* = 25.9), which is the highest in all SBS signatures (Fig. 2*K*).

The SBS11-like C>T mutations are largely eliminated when the templates are treated with human methylguanine methyltransferase (hMGMT), which can remove the methyl group from O^6^-meG (Fig. 2, *C* and *H*). This indicates that O^6^-meG modification was the major cause of the TMZ-induced C>T mutations. A single round of TMZ exposure did not produce an SBS11-like spectrum (Fig. 2*F*). Instead, a distinct pattern of mutations was observed, including misincorporations of dAMP at C residues (resulting in G>A mutations) on damaged templates (Fig. 2*F*, *blue bars* in the C→T section). After subsequent exposures, these G>A mutations were masked by the emergence of predominating C>T mutations at G residues. Although the underlying reason remains unclear, the disappearance of the G>A mutations after repeated TMZ exposures was reproducible in independent experiments (Fig. S3). The SBS11-like spectrum was not observed when ssDNA templates were used (Fig. 2*I*). Instead, G>A mutations on C residues were observed on the ssDNA templates even after multiple TMZ exposures.

### DNA polymerases mediate mutagenic and nonmutagenic translesion synthesis across defined O^6^-meG

As shown above, TMZ-induced SBS11-like mutations were mainly produced at O^6^-meG lesions of the templates by yPol δ *in vitro*. To confirm the mutagenic translesion synthesis (TLS) activity of yPol δ and to investigate the behaviors of other DNA polymerases, we analyzed the TLS activities of various DNA polymerases across a defined O^6^-meG residue (Fig. 3). Consistent with their high fidelity, yPol δ and yeast Pol ε (yPol ε) showed low efficiency in bypassing the O^6^-meG, but the full-length products were observed after 40 min of incubation under our experimental conditions (Fig. 3*B*). Human Pol η (hPol η), yeast Pol ζ (yPol ζ), and human Pol κ (hPol κ) demonstrated more efficient TLS across the lesion.

NGS-based sequencing of the TLS products (Fig. 3*C*) showed that the polymerases inserted the correct nucleotide (dCTP) at the defined O^6^-meG with varying frequencies, indicating their varying accuracies in TLS. Among them, hPol η showed the highest accuracy (51%), whereas yPol ζ exhibited the lowest (4.5%). Analysis of mutation types in the mutated products (Fig. 3*D*) showed that more than 70% of the mutations produced by any tested polymerase were misincorporations of dTMP, causing C>T mutations. Two Y-family polymerases, hPol η and hPol κ, showed both high TLS activity and accuracy, confirming their roles in nonmutagenic TLS. When these polymerases were combined with yPol δ, the amount and accuracy of the TLS products were not greatly altered. However, dGMP misincorporation by yPol ζ was reduced in the presence of yPol δ, suggesting that dGMP, compared with dTMP, is more readily removed by the proofreading activity of yPol δ.

### hPol η suppresses TMZ-induced SBS11-like mutations *in vitro*

**Figure 4:**
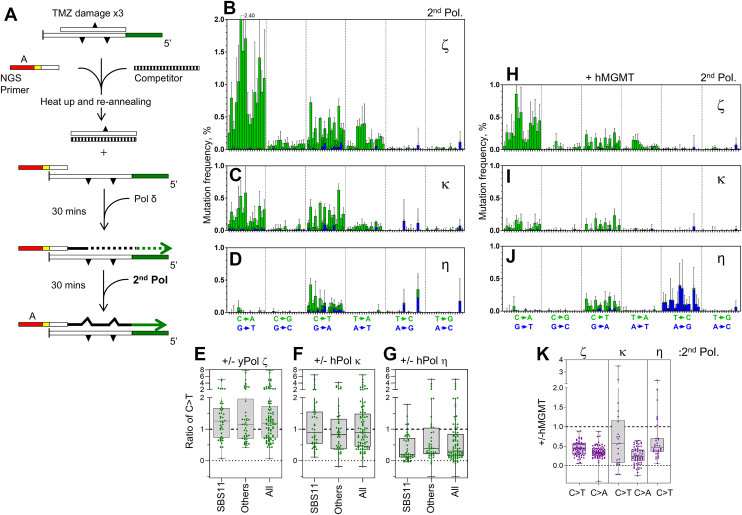
**Influence of TLS polymerases on the TMZ-induced mutation spectra in the presence and absence of hMGMT.***A,* illustration of the experimental system. dsDNA substrates were incubated with 400 μM TMZ three times. When indicated, damaged templates were treated with hMGMT. The DNA was heated and reannealed with NGS primer and 10x excess competitor to sequester the top strand. Then the primer extension was started by adding yPol δ. After 30 min of incubation with yPol δ, the second polymerase (either yPol ζ, hPol κ, or hPol η) was added, and the reaction was continued for another 30 min. *B*–*D,* mutation spectra produced on the TMZ-damaged DNA in the presence of the indicated second polymerase without hMGMT treatment. *E–G,* influences of the second polymerase on the C>T mutations were expressed as a ratio of the mutation frequencies at individual sites. CpC>T and CpT>T mutations (SBS11), other mutations (Others), and all mutations (All) are plotted as separate groups. *H*–*J,* the same experiments as in *B*–*D* were carried out using the templates that were treated with hMGMT. *K,* influences of hMGMT on the C>T and C>A mutations that were produced in the presence of indicated second polymerases. For hPol η reactions, only C>T mutations were analyzed because this polymerase did not produce considerable C>A mutations. Mutation frequencies mapped on the templates are shown in Fig. S4. hMGMT, human methylguanine methyltransferase; hPol κ, human Pol κ; hPol η, human Pol η; NGS, next-generation sequencing; SBS11, substitution signature 11; TLS, translesion synthesis; TMZ, temozolomide; yPol δ, yeast Pol δ.

While the SBS11-like mutation spectrum was produced by yPol δ alone (Fig. 2*G*), hPol η, hPol κ, and yPol ζ exhibited higher activities to bypass O^6^-meG. Since hPol η and hPol κ showed higher accuracy in TLS across O^6^-meG compared with the replicative polymerases, they may decrease TMZ-induced mutation frequency. In addition, these TLS polymerases may produce distinct mutation spectra on TMZ-damaged DNA. Thus, we analyzed the influences of hPol η, hPol κ, and yPol ζ on TMZ-induced mutation spectra (Fig. 4). Because these TLS polymerases are error-prone polymerases that make high background mutations on undamaged templates, we combined them with yPol δ to minimize the background mutations (Fig. 4*A*). The *in vitro* mutation spectra produced in the presence of both yPol δ and one of the TLS polymerases (yPol ζ, hPol κ, or hPol η) on TMZ-damaged dsDNA are shown in Figure 4, *B*–*D*. Comparison of these spectra with the one produced by the yPol δ-only reaction (Fig. 2*G*) highlighted two remarkable changes that were induced by the TLS polymerases. First, as predicted, hPol η reduced C>T mutations by approximately 70% (Fig. 4, *D* and *G*), confirming its function as a suppressor of TMZ-induced mutagenesis. Second, prominent C>A mutations were produced by yPol ζ and hPol κ on G residues of the templates (*green bars* in the left-most sections of Fig. 4, *B* and *C*). hMGMT treatment reduced the C>A mutations to less than 50% compared with no hMGMT treatment (Fig. 4, *H*, *I*, and *K*). However, it is unlikely that the C>A mutations were produced by O^6^-meG because yPol ζ and hPol κ rarely inserted dAMP at defined O^6^-meG (Fig. 3*D*). This study could not clearly identify the damage that caused C>A mutations.

**Figure 5:**
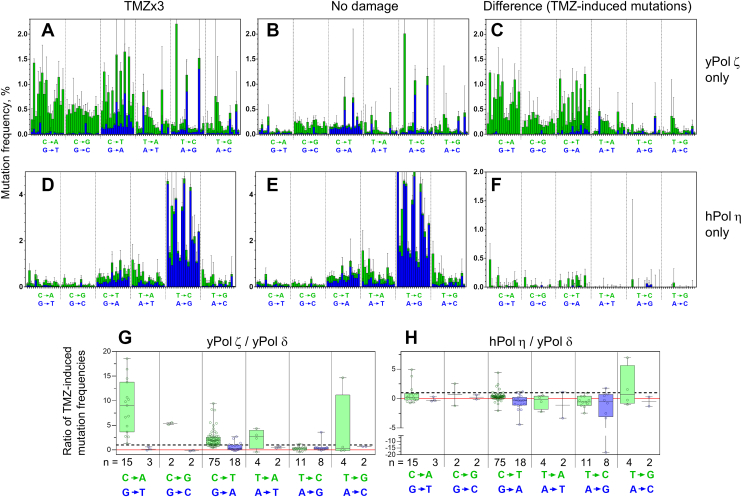
**TMZ-induced mutations by yPol ζ and hPol η in single polymerase reactions.** Trinucleotide mutation spectra by yPol ζ (*A* and *B*) or by hPol η (*D* and *E*) on the TMZ-damaged (*A*, *D*) or undamaged (*B*, *E*) templates were computed without background subtraction. Note that these spectra include mutations that were made by the polymerases on undamaged residues and by pre-existing errors in the templates. *C, F,* TMZ-induced mutations by yPol ζ (*C*) and by hPol η (*F*) are obtained by subtracting data in *B* and *E* from *A* and *D*, respectively. *G,* frequencies of yPol ζ-mediated and TMZ-induced mutations (shown in *C*) are divided by those mediated by yPol δ on the same template (shown in Fig. 2*G*) at individual residues. The ratios are calculated at all residues at which the mutation frequencies by yPol δ are higher than 0.05%. Numbers of qualified residues (n) are shown under the graph. The *lateral dashed line* and *red line* indicate *y* = 1 and 0, respectively. *H,* the same analysis as in *G* is applied for hPol η-mediated mutations (shown in *F*). hPol η, human Pol η; TMZ, temozolomide; yPol ζ, yeast Pol δ.

While combinations of polymerases can reduce background mutations, the roles of each polymerase during the TLS process cannot be readily interpreted from the data. To clarify the roles of each TLS polymerase, single polymerase reactions were carried out by yPol ζ and hPol η on the TMZ-damaged DNA (Fig. 5, *A* and *D*) and on undamaged DNA (Fig. 5, *B* and *E*). Although yPol ζ produced considerable amounts of mutations on undamaged DNA, TMZ treatment clearly increased mutation frequencies. After subtracting the background, TMZ-induced mutation frequency by yPol ζ showed similar patterns of C>A and C>T mutations (compare Figs. 5*C* and 4*B*), indicating that these mutations can be produced by yPol ζ alone. yPol ζ also introduced C>G and T>A mutations on the TMZ-damaged templates at lower frequencies. In contrast, hPol η-mediated mutation spectra were not greatly changed in the presence or absence of TMZ treatment (Fig. 5, *D* and *E*). The TMZ-induced portion (Fig. 5*F*) showed lower frequencies of C>A and C>T mutations than Figure 4*B*, confirming accurate TLS by hPol η across the damaged G residues. Interestingly, hPol η produced lower frequencies in all types of TMZ-induced mutations than yPol δ (Fig. 5*H*). This suggests that the accuracy of TLS by hPol η is higher than that of yPol δ at multiple types of damages produced by TMZ. However, because of the high frequencies of background mutations by hPol η, damage-induced mutation frequencies by the error-prone polymerases are difficult to evaluate.

## Discussion

In this article, we reconstituted TMZ-induced mutational processes in a cell-free system. Under controlled biochemical conditions, we explored the essential reactions to produce mutations that had a similar pattern to SBS11, a hallmark of TMZ-treated glioblastomas. Our *in vitro* experiments reproduced the mutations that had a similar trinucleotide context to SBS11, including C>T transitions with CpC and CpT contexts. Enzymatic activation of TMZ or other cellular events like DNA repair or nucleosome formation were not included in the *in vitro* system; thus, they seem to be nonessential for mutagenic processes that produce SBS11.

### O^6^-meG modification and translesion DNA synthesis by replicative polymerases are involved in an essential process in SBS11 generation

Our *in vitro* results showed SBS11-like mutations occurred only on G residues of the templates, and they were eliminated by MGMT. Since MGMT specifically removes methyl groups from oxygen outside the purine/pyrimidine rings (36), this result indicates that O^6^-meG is directly involved in the production of SBS11-like mutations *in vitro* (Fig. 3). The implication of O^6^-meG in TMZ-induced mutagenesis is supported by previous observations showing that MGMT promoter methylation and MGMT activity are related to TMZ resistance (37, 38). It has also been reported that O^6^-meG is the major damage responsible for cytotoxic effects (8, 12, 31, 39). Therefore, this lesion is crucial for both cytotoxicity and genotoxicity by TMZ. Our data also show that the replicative polymerase yPol δ can produce SBS11-like mutations on TMZ-damaged DNA. Although human replicative polymerases may behave differently at O^6^-meG, our results suggest that ordinary DNA replication may be sufficient to drive this mutagenic process. From the therapeutic perspective, this highlights the difficulty in controlling the TMZ activity. O^6^-meG is responsible for both the therapeutic efficacy and the genotoxic side effects of the treatment. In addition, the most fundamental function of DNA replication may be directly involved in the mutagenic process.

### Mutational contributions of TLS polymerases

We also analyzed the roles of TLS polymerases hPol η, hPol κ, and yPol ζ in the damage bypass and mutagenesis. These polymerases demonstrated higher activities in bypassing defined O^6^-meG lesions (Fig. 3) and exhibited distinct mutation spectra on TMZ-damaged templates (Figs. 4 and 5). Notably, yPol ζ and hPol κ introduced C>A mutations that were not associated with SBS11. The precise nature of the C>A mutations remains unclear. The mutations were partially sensitive to MGMT treatment, suggesting that O^6^-meG contributes to at least a subset of C>A mutations. However, this interpretation is inconsistent with the results in Figure 3*D*, where the TLS polymerases did not efficiently insert dAMP at the defined O^6^-meG. This discrepancy may highlight the complexity of the mutagenic mechanisms. One possibility is that these polymerases may insert dAMP at O^6^-meG that has additional DNA damage at close proximity. Further studies are required to understand the influence of neighboring lesions on the mutagenicity of the TLS polymerases. In addition, the mutagenic TLS activities of yPol ζ and hPol κ have been implicated in TMZ-resistant cell lines (3). Evaluating the roles of these TLS polymerases within the DNA damage response network to TMZ is critical for understanding resistance mechanisms. Remarkably, hPol η showed no C>A mutation and very low frequencies of C>T mutations on the TMZ-damaged templates. This result provides additional evidence to support the suppressive role of this polymerase upon DNA damage. hPol η also induced A>G mutations on T residues of the templates (Fig. 4*J*). However, the A>G mutations are likely because of damage-independent dGMP misincorporations at T residues by hPol η (34), which is also observed in Figure 5*E*.

### Dynamic nature of DNA damages during repeated exposures

Production of SBS11-like mutations *in vitro* required repeated exposures to TMZ. Single exposure to TMZ preferentially induced G>A mutations, which occurred on C residues of the templates. This suggests that TMZ modified C residues during the first exposure, then modified G residues, producing O^6^-meG, during subsequent exposures (Figs. 2, *F* and *G* and S3). The nature of C damages and the mechanism of damage switching is not clear at this point. Previous studies using *in vitro* polymerase assays have shown that 5-meC modifications reduce DNA extension rates (40) and have proposed that 5-meC can mispair with adenine, leading to G>A mutations, particularly in GCG contexts (41, 42). The G>A mutations in our results might be caused by mispairing of 5-meC with A, but we cannot explain why the G>A mutations were reduced after repeated exposures.

Interestingly, Stojic *et al.* (43) reported that another SN1 alkylator, methylnitronitrosoguanidine, induced single-strand DNA breaks or gaps independent of the MMR machinery in 293T Lα cells. These breaks and gaps were observed to be resolved over time (43), suggesting that some initial damages may be unstable or cause polymerase-induced breaks or gaps. In the context of our study, this raises the possibility that the increase in damage because of subsequent exposures could similarly destabilize replication polymerase activity at me-C sites, potentially increasing the likelihood of such breaks or gaps. If gaps are indeed generated with increased exposures, they would not be captured by our system, which detects only full-length readouts, and could explain the observed disappearance of G>A mutations. Together, these observations suggest a potential link between neighboring damage, me-C instability, and the dynamics of G>A mutation disappearance, warranting further evaluation. Understanding the enzymatic and structural factors that dictate these mutational outcomes can mitigate resistance by combining strategies of TMZ therapy along with modulating the activity of DNA damage response enzymes.

### Conditions for the polymerase reactions

The standard condition for DNA polymerase reactions includes 100 μM of four dNTPs, 5 mM of MgCl_2_, and 50 mM of NaCl (see the *Experimental procedures* section). These conditions were established after optimization experiments, during which we observed that individual polymerases have different optimum conditions. For example, yPol ζ prefers a much higher (150–200 mM) NaCl concentration than yPol δ (30–50 mM), and hPol κ has a 30-fold lower *K*_*m*_ (0.3 μM) for dNTP incorporations than yPol δ (11 μM). Under the standard conditions, all polymerases showed at least 50% of their maximum activities. Nonetheless, biochemically optimized reactions might not faithfully reflect *in vivo* situations. In particular, lower dNTP concentrations may influence mutation frequencies and spectra. We did not extensively analyze the mutation spectra with different ranges of dNTP concentrations. When we analyzed mutation frequencies in more physiologically relevant conditions (150 mM NaCl, 1 mM MgCl_2_, 24 μM dATP, 5.2 μM dGTP, 29 μM dCTP, and 37 μM dTTP), the reaction did not produce sufficient primer-extension products by yPol δ (Fig. S5).

Although an SBS11-like mutation spectrum was successfully produced by yPol δ, human Pol δ may behave differently at TMZ-damaged DNA. However, a recent work shows that human Pol δ can efficiently bypass 6-meG (44), supporting the idea that human Pol δ is also involved in the TMZ-induced mutagenesis and carcinogenesis. Other protein factors that were not examined in this article, including proliferating cell nuclear antigen and replication protein A, potentially influence mutation frequency and spectrum *in vitro*. Especially, it is interesting to examine the function of proliferating cell nuclear antigen modifications on the mutation spectra.

## Experimental procedures

### DNA and proteins

Sequences of synthetic DNA templates and the top strands used in this study are shown in Table 1 and Fig. S1. All synthetic DNAs were purchased from Integrated DNA Technologies, except for the template A2, which was purchased from Sigma–Aldrich. The template DNA had the same sequences as those previously reported (template A–G (32, 33, 34, 35)), with the only change that new templates had a different primer-hybridization sequence (nucleotide position #1–20 in Fig. S1). The dsDNA templates were created by annealing ssDNA templates with their complementary oligos (“top strand” for each template, Table 1 and Fig. 1*A*). The top strand is complementary to the variable region (Fig. 1, *A* and *B*). The template with defined O^6^-meG residue (used in Fig. 4) was purchased from Integrated DNA Technologies. Exact sequences are shown in Fig. S1*B*.

**Table 1:** Sequences of DNA templates (A2–G2) and the top strands (Top-A2 to Top-G2)

Template	Sequence
A2	CCTCTCTATGGGCAGTCGGTGATGACTAGATATCGACGTGATCAGACTCTAGATAGATGCTAGAGAGCTCTATCGATCATCCTTGTTACTGCCTCACTC[BtnTg]
B2	CCTCTCTATGGGCAGTCGGTGATGACGTGTGACTGCATACATAGCTACACTGAGTATACGATCGCTAGCATCGTGATCATCCTTGTTACTGCCTCACTC/3Bio/
C2	CCTCTCTATGGGCAGTCGGTGATCATCGTCTGCTCATGTAGACGCGCTGTCTCGATGTATACGACAGTCAGACTGCTCATCCTTGTTACTGCCTCACTC/3Bio/
D2	CCTCTCTATGGGCAGTCGGTGATACGCACACAGTACATGCGCGACGTGACGACAGTCGACACATGTAGATCGCTCGTCATCCTTGTTACTGCCTCACTC/3Bio/
E2	CCTCTCTATGGGCAGTCGGTGATGCTAAACGGGCTTCCCTAGGACCAATTAAGTTTGCCTGGAACCGGTCTTCACGTCATCCTTGTTACTGCCTCACTC/3Bio/
F2	CCTCTCTATGGGCAGTCGGTGATAACCGGATTCCCTTAAGGGTTCAACCAGGCCTGGAAAGGCAATTTGGTTACCGTCATCCTTGTTACTGCCTCACTC/3Bio/
G2	CCTCTCTATGGGCAGTCGGTGATACCCTTGGGCCAATCCCTTTGGTTAAACGCCAGCGAATTTCCGGGAAAGGAGTTCATCCTTGTTACTGCCTCACTC/3Bio/


Top strand	Sequence
Top-A2	GATCGATAGAGCTCTCTAGCATCTATCTAGAGTCTGATCACGTCGATATCTAGTCAT
Top-B2	GATCACGATGCTAGCGATCGTATACTCAGTGTAGCTATGTATGCAGTCACACGTCAT
Top-C2	GAGCAGTCTGACTGTCGTATACATCGAGACAGCGCGTCTACATGAGCAGACGATGAT
Top-D2	GACGAGCGATCTACATGTGTCGACTGTCGTCACGTCGCGCATGTACTGTGTGCGTAT
Top-E2	GACGTGAAGACCGGTTCCAGGCAAACTTAATTGGTCCTAGGGAAGCCCGTTTAGCAT
Top-F2	GACGGTAACCAAATTGCCTTTCCAGGCCTGGTTGAACCCTTAAGGGAATCCGGTTAT
Top-G2	GAACTCCTTTCCCGGAAATTCGCTGGCGTTTAACCAAAGGGATTGGCCCAAGGGTAT

The sequences of the top strand are complementary to the respective variable regions of individual templates.

yPol δ (complex of Pol3–Pol31–Pol32 subunits) with His6-tag at C terminus of Pol32, yPol ε (catalytic subunit) with His6-tag at C terminus (34), yPol ζ (complex of Rev3–Rev7–Pol31–Pol32 subunits) in which Rev7 and Pol32 were tagged with FLAG and His6, respectively, hPol η with His6-tag at C terminus, hPol κ with C-terminal His6-tag were purified in our previous works (34, 45). Concentrations of the polymerases were adjusted to 200 nM with 30 mM Tris–HCl (pH 7.5), 50 mM NaCl, 1 mM EDTA, 1 mM DTT, and 5% glycerol and stored at −80 °C until 3 to 5 min before adding to the reaction. Human complementary DNA of MGMT (hMGMT) was obtained from SinoBiological (46), amplified by PCR, and cloned into pET21a to express MGMT with a C-terminal His6-tag. After confirming the DNA sequence, the plasmid was introduced into *Escherichia coli* BLR(DE3) harboring the pCodonPlus plasmid (Agilent Technologies) for overexpression of the protein. Protein expression was induced by IPTG for 15 to 16 h at 18 °C. The cells were harvested by centrifugation and stored at −80 °C. For purification, frozen cells were thawed on ice and suspended in the lysis buffer (50 mM Tris–HCl [pH 7.5], 5% [vol/vol] glycerol, 1 M NaCl, and 10 mM imidazole) supplemented with 1 mM PMSF. All the purification procedures afterward were carried out on ice or at 4 °C as previously published (46). Q-Sepharose (Cytiva) was used as the second column for hMGMT purification. hMGMT concentration was determined by the band intensity of the protein after SDS-PAGE compared with the bovine serum albumin standard (Pierce).

### TMZ treatment

TMZ was purchased from Sigma–Aldrich (47) and dissolved in dimethyl sulfoxide to make a 1 M stock, which was stored at −20 ˚C. Just before use, the TMZ stock solution (1 M) was diluted with Tris–HCl pH buffer (pH 7.5) and incubated for 10 min at 37 ˚C. Then, a DNA damage reaction (200 μl) was carried out by incubating 200 nM dsDNA and 400 μM TMZ in 50 mM Tris–HCl (pH 7.5) buffer for 1.5 h (unless otherwise stated) at 37 °C. For conducting repeated exposures of TMZ, 400 μM of TMZ was repeatedly added at an interval of 1.5 h. After TMZ exposure, DNA was precipitated with ethanol and resuspended in H_2_O. To treat seven templates (template #A2–#G2) under the same conditions, they were pooled in a single tube to make a final 200 nM of total DNA and incubated with TMZ repeatedly as described previously. When indicated, TMZ-treated templates were also treated with 400 nM of hMGMT for 1 h in 5 mM T–s–HCl (pH 8.0), 1 mM EDTA, and 5 mM DTT (48, 49) at 37 °C, then purified by MicroSpin G-25 Columns (Cytiva), deproteinized by phenol–chloroform extraction, precipitated with ethanol, and resuspended in H_2_O.

### Primer extension

Primer extension from ^32^P-labeled primer was carried out as previously published (32, 33, 34, 35). In a standard reaction (10 μl), DNA template (0.11 pmol) and ^32^P-labeled 23-mer primer (0.1 pmol) were annealed by heating to 94 °C for 4 s and cooling to 37 °C over 30 min, and yPol δ (0.2 pmol in 1 μl) was added to start primer extension. The standard reaction buffer at this point contained 25 mM Tris–acetate (pH 7.5), 50 mM NaCl, 4 mM MgCl_2_, 100 μg/ml bovine serum albumin, 5 mM DTT, and 100 μM of each of the four dNTPs. After incubating for 40 min at 37 °C, the reaction was stopped by mixing 1.5-fold of stop buffer (20 mM EDTA, 0.1% bromophenol blue, and 0.1% xylenecyanol in formamide). Labeled DNA products were analyzed by 10% polyacrylamide gel electrophoresis, and ^32^P-labeled DNA products were visualized and quantified with a Bio-Rad Molecular Imager Personal FX and Quantity One Software. When a single polymerase was used in a single reaction, the total reaction time was 40 min. When two polymerases were used in a single reaction, DNA was incubated first with yPol δ (0.2 pmol in 1 μl) for 30 min. and then the second polymerase (0.2 pmol in 1 μl) was added and incubated for another 30 min (1 h total reaction time). In reactions with defined O^6^-meG damage, the second polymerase was added at 15 min and incubated for another 25 min (40 min reaction time).

The primer-extension reaction for mutation assay was carried out under the same conditions as aforementioned, except that NGS primers (Fig. 1*B*) were used, and the reaction was stopped by phenol–chloroform–isoamyl alcohol extraction, then DNA was precipitated with ethanol, and dissolved in 10 μl H_2_O. Multiple tractions were carried out with primers containing distinct barcodes, and samples were pooled and analyzed with Ion Chef and Ion S5 sequencing system (Thermo Fisher Scientific).

### Data analysis

Detailed procedures for quantification and analysis of NGS data have been published previously (32, 33, 34, 35). Briefly, sequence reads of full-length extension products were subjected to Lastz 1.3.3 sequence alignment tool to detect base alteration against the reference sequences of each template. After that, sequences of primer extension products, not template extension products, were selected by selecting reads that had a mismatch at the center of the primer-template hybridization site (Fig. 1. E, “10G”). Total numbers of the qualified reads are summarized in Table 1. Substitution frequencies of each base of the templates (a total of 350-nts) were calculated from the numbers of given mutations at each position of the templates and the numbers of qualified reads that had the 10G. Background subtraction was conducted by using the substitution frequencies that were obtained on the same templates without TMZ exposure. Trinucleotide mutation spectra were computed as described (32, 34). For comparison, COSMIC signatures (version 3.2) were downloaded from https://cancer.sanger.ac.uk/cosmic/signatures.

## Data availability

Data will be made available on request.

## Supporting information

This article contains supporting information.

## Conflict of interest

The authors declare that they have no conflicts of interest with the contents of this article.
