# Supplementary material for: Biochemical reconstitution of temozolomide-induced mutational processes
Source: J Biol Chem. 2025 Sep 4;301(11):110676. doi: 10.1016/j.jbc.2025.110676 (PMC12556801; doi:10.1016/j.jbc.2025.110676)
Supplement: Supplementary Data [file mmc1.pdf]

Title

Biochemical reconstitution of temozolomide-induced mutational processes

Authors

Mahima R. Sanyal[1,2] and Tomohiko Sugiyama[1,2,*]

Department, University

[1]Department of Biological Sciences, Ohio University, Athens, Ohio, United States of America

[2]Molecular and Cellular Biology Graduate Program, Ohio University, Athens, Ohio, United States of America

Corresponding author

Email: sugiyama@ohio.edu

List of Figures included:

1. Fig S-1
2. Fig S-2
3. Fig S-3
4. Fig S-4
5. Fig S-5

List of Tables included:

1. Table S-1

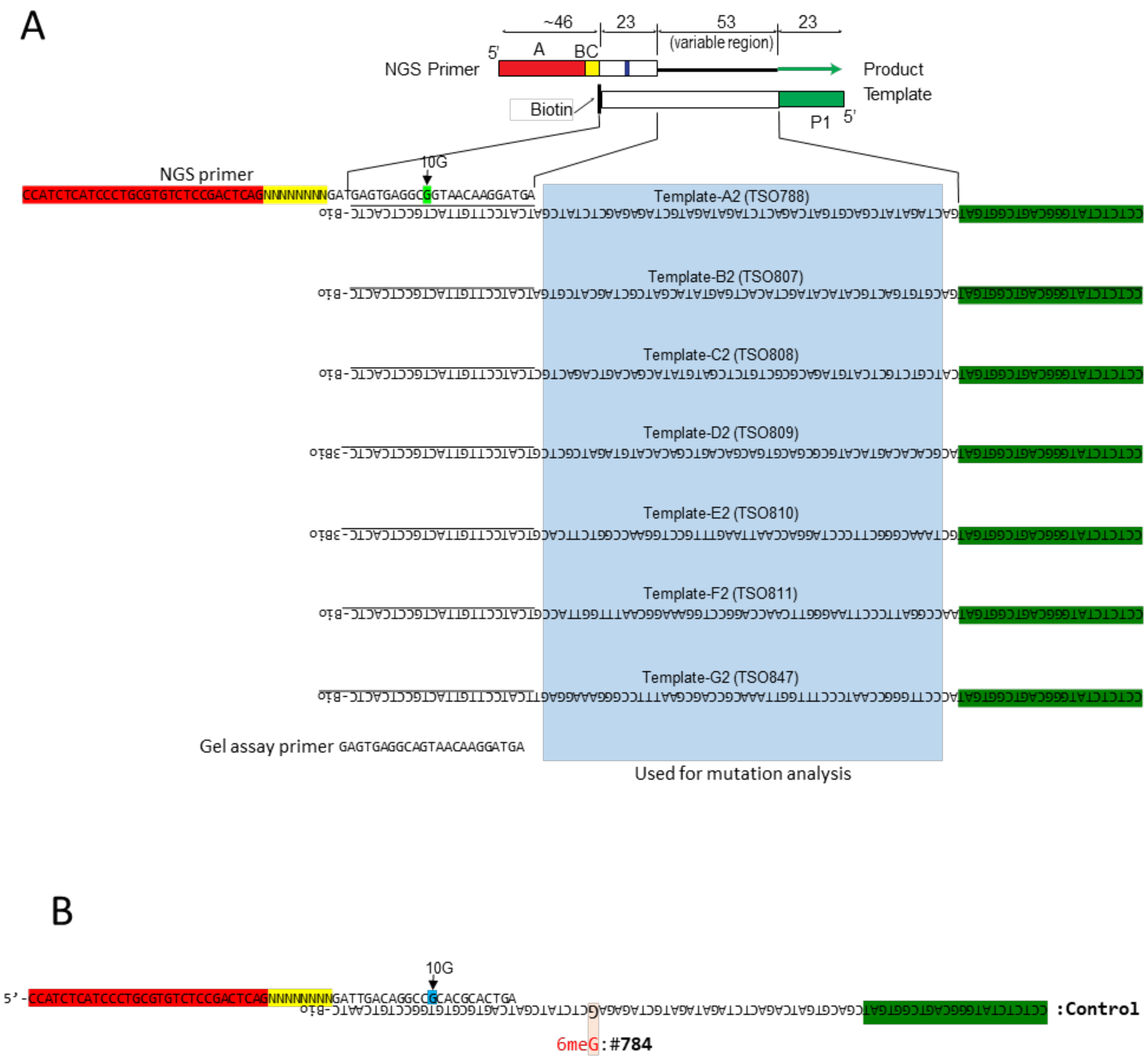

Figure S1. *A,* Sequences of synthetic ssDNA templates (templates A2-G2) and a NGS primer. *B,* Synthetic DNA containing O6-meG residue at defined position.

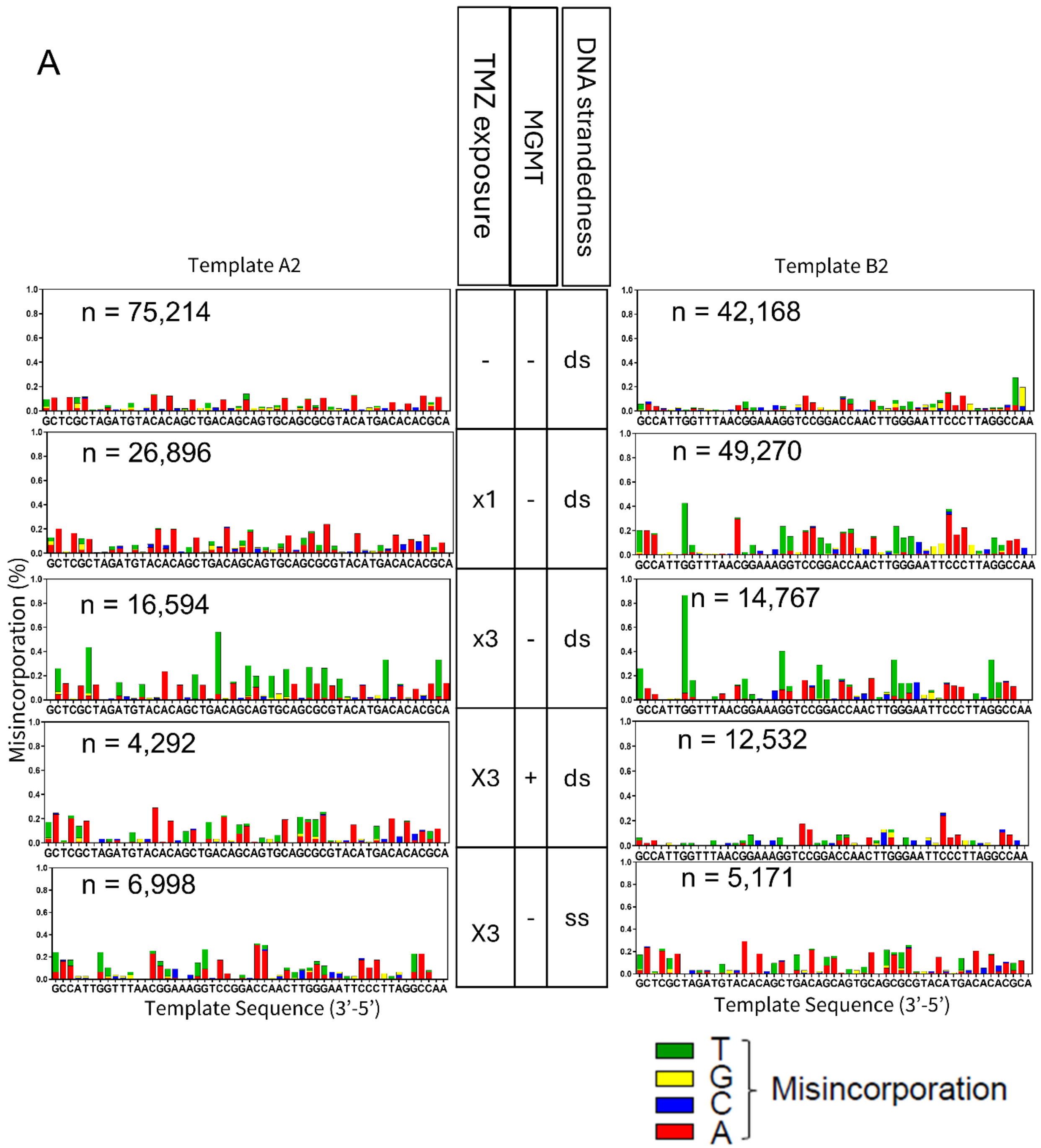

Figure S2.*A* TMZ-induced mutation analysis. Single-nucleotide substitution frequencies of TMZ damaged templates (A2 and B2) were quantified and mapped on individual template sequences that are shown in Fig S1A. Different conditions such as TMZ exposures, MGMT treatment and DNA strandedness have been indicated.

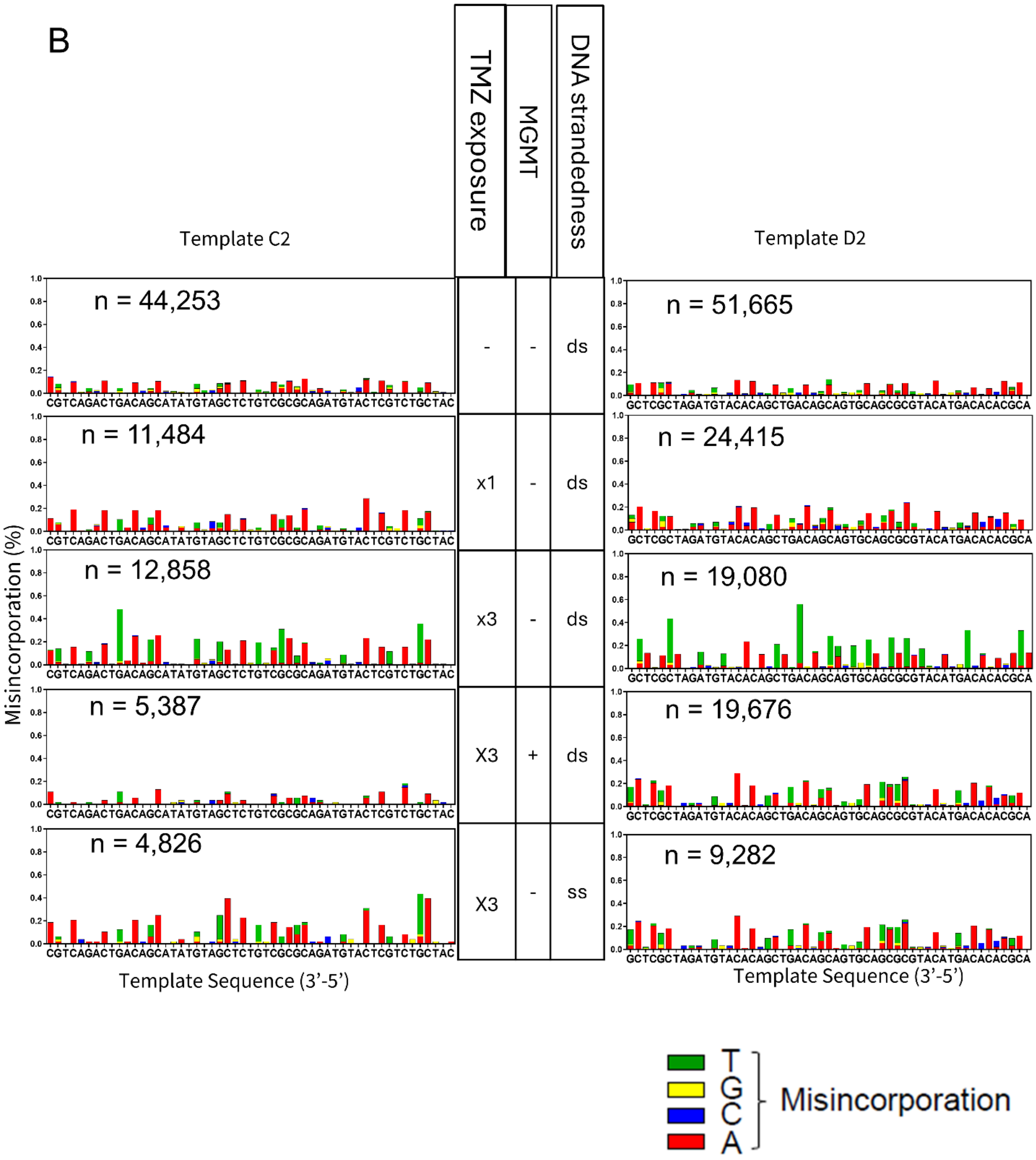

TMZ exposure	MGMT	DNA strandedness
-	-	ds
x1	-	ds
x3	-	ds
X3	+	ds
X3	-	ss

Figure S2.*B* TMZ-induced mutation analysis. Single-nucleotide substitution frequencies of TMZ damaged templates (C2 and D2) were quantified and mapped on individual template sequences that are shown in Fig S1A. Different conditions such as TMZ exposures, MGMT treatment and DNA strandedness have been indicated.

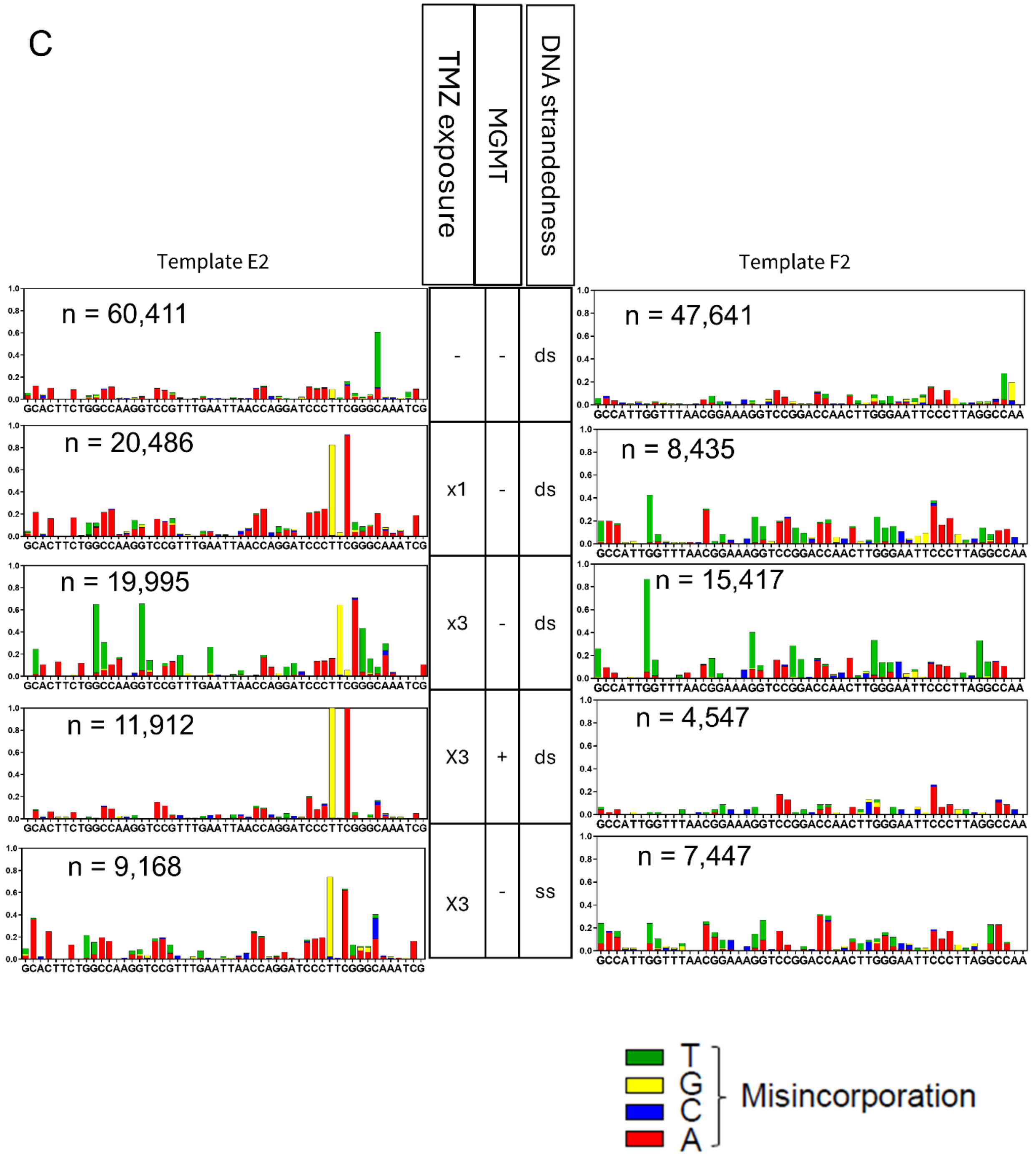

Figure S2.*C* TMZ-induced mutation analysis. Single-nucleotide substitution frequencies of TMZ damaged templates (E2 and F2) were quantified and mapped on individual template sequences that are shown in Fig S1A. Different conditions such as TMZ exposures, MGMT treatment and DNA strandedness have been indicated.

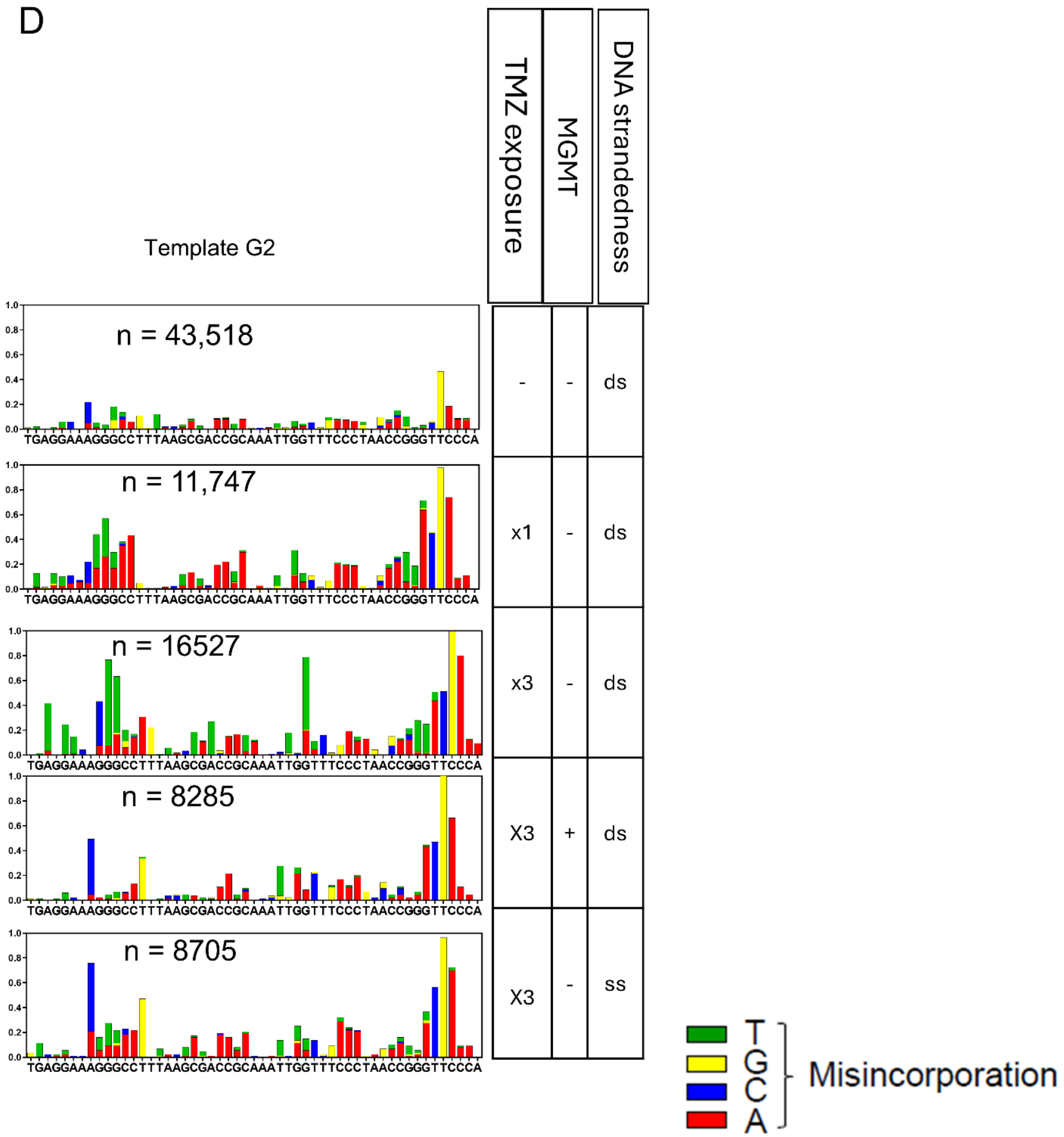

Figure S2.*D* TMZ-induced mutation analysis. Single-nucleotide substitution frequencies of TMZ damaged templates (G2) were quantified and mapped on individual template sequences that are shown in Fig S1A. Different conditions such as TMZ exposures, MGMT treatment and DNA strandedness have been indicated.

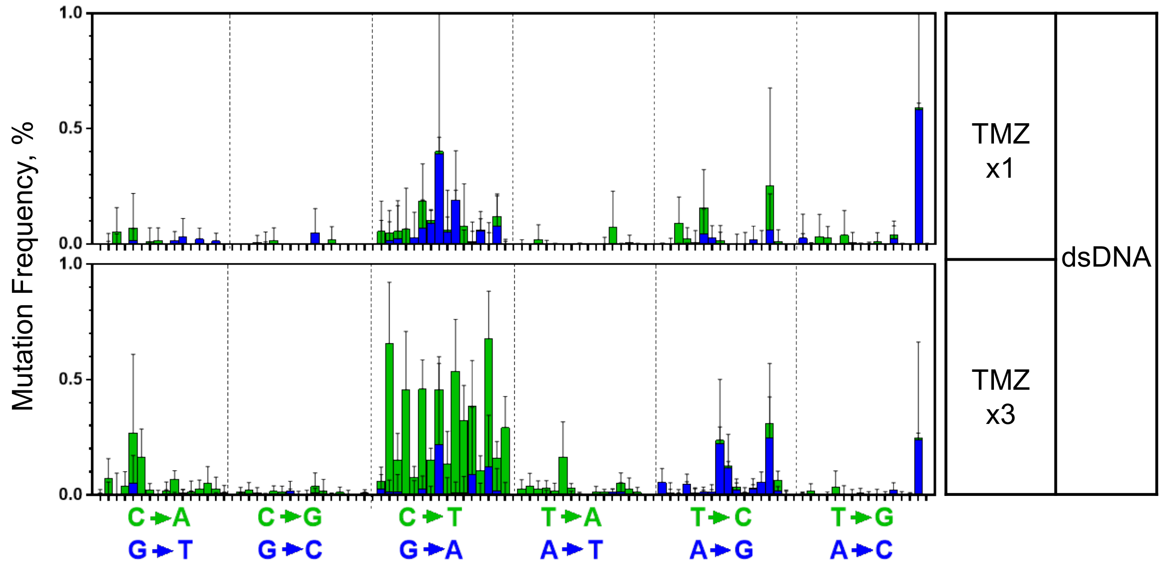

Figure S3. Experiments shown in Fig. 2F and G are independently repeated. Note that the G>A mutations were reduced while C>T mutations newly appeared after the repeated exposures.

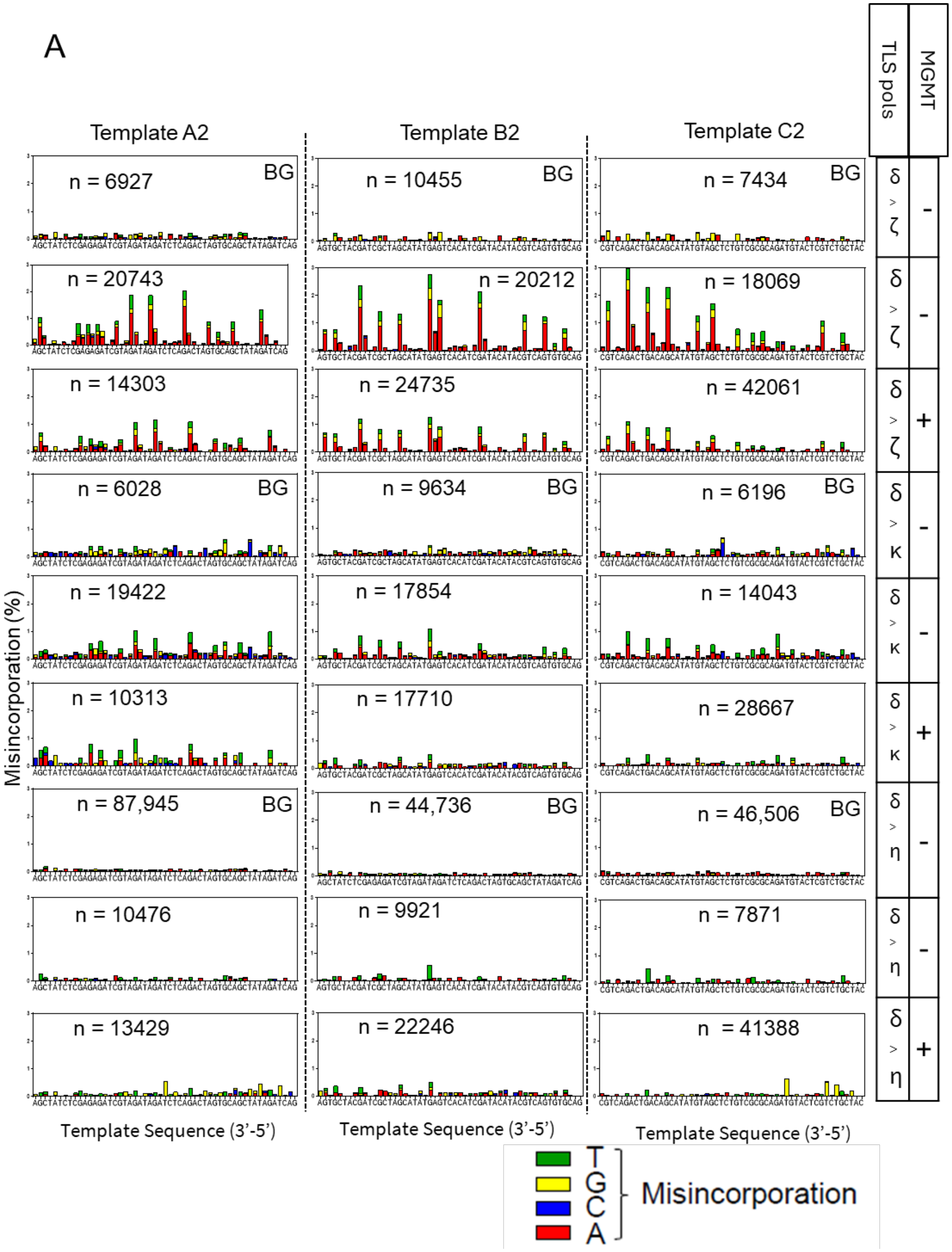

Figure S4 ***A:*** Single-nucleotide substitutions by yPol δ and Pols κ,η or ζ on the 3x TMZ-damaged templates (A2-C2) in the presence or absence of hMGMT treatment. BG: mutations without TMZ treatment.

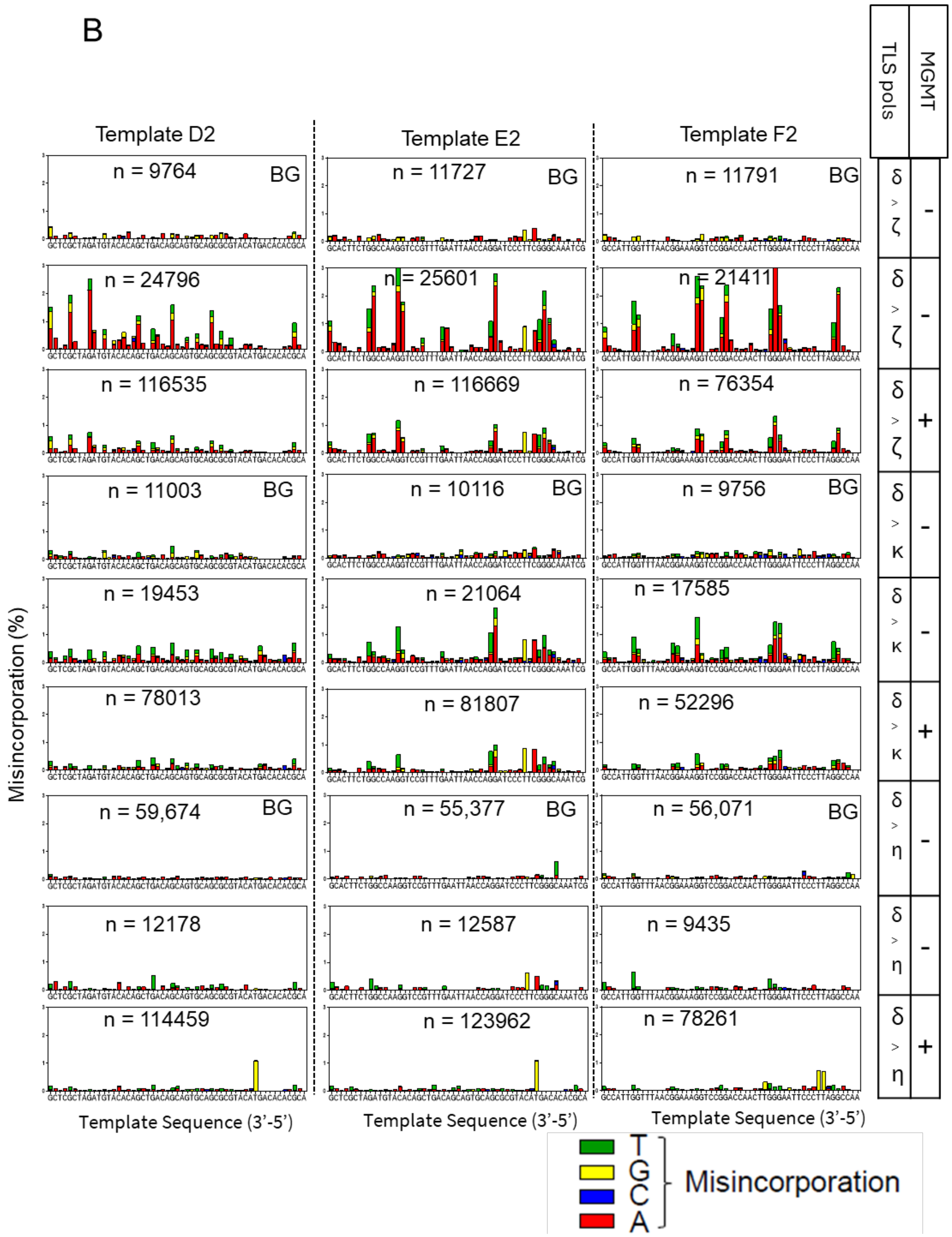

Figure S4 *B:* Single-nucleotide substitutions by yPol δ and Pols κ,η or ζ on the 3x TMZ-damaged templates (D2-F2) in the presence or absence of hMGMT treatment. BG: mutations without TMZ treatment.

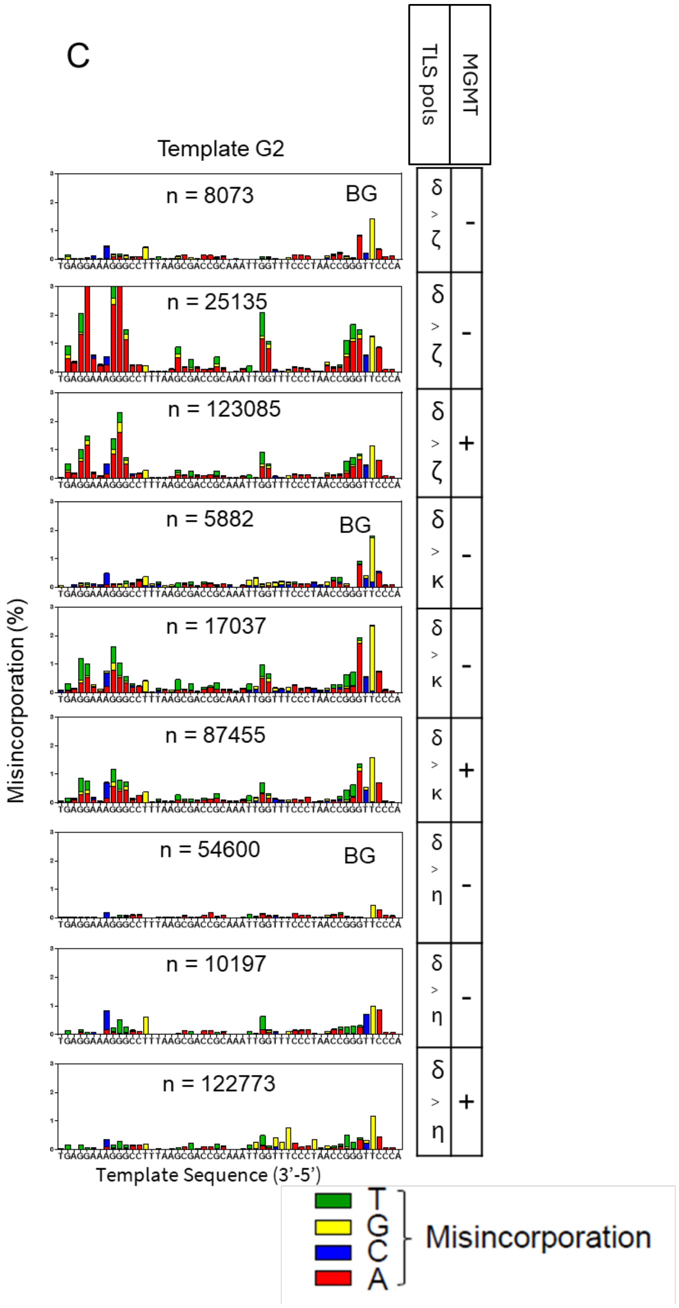

Figure S4 ***C:*** Single-nucleotide substitutions by yPol δ and Pols κ,η or ζ on the 3x TMZ-damaged template (G2) in the presence or absence of hMGMT treatment. BG: mutations without TMZ treatment.

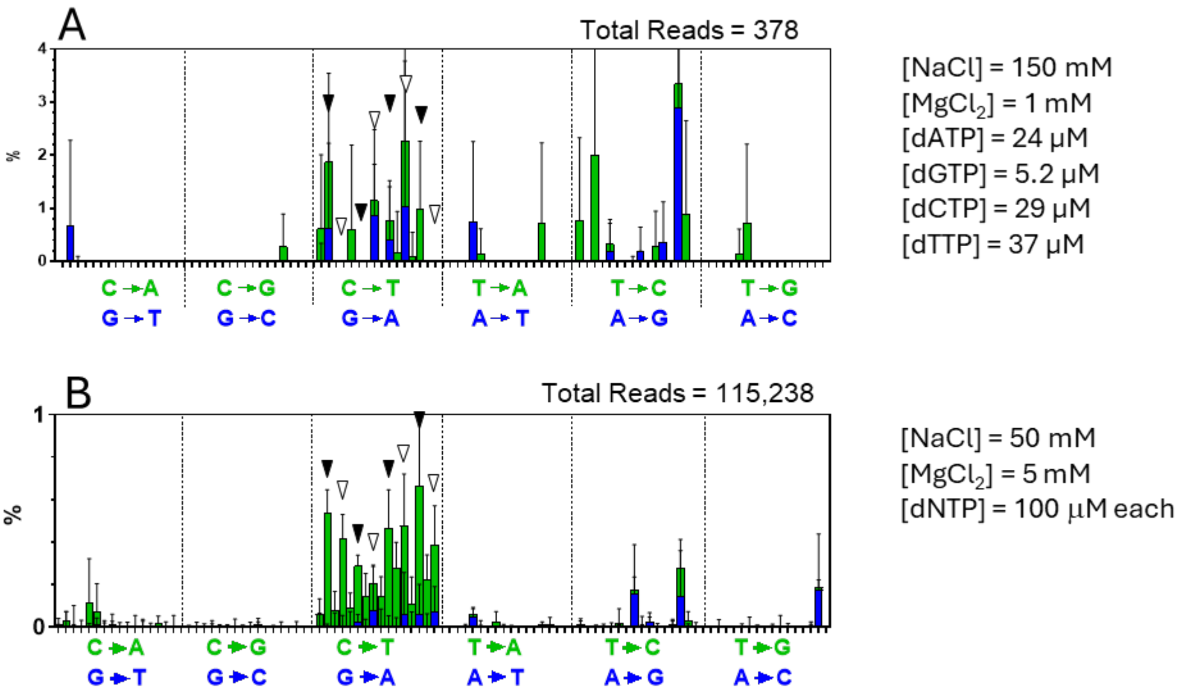

Figure S5. ***A***, Mutation spectrum of TMZ-damaged DNA, produced by yPol d under more physiological primer extension conditions. The experiment as in Fig. 2G was repeated under the same conditions except that the primer extension reaction contained indicated concentrations of NaCl, $MgCl_2$, and dNTPs. ***B***, Fig. 2G is copied for comparison. Note that the total qualified reads of panel ***A*** was much fewer (~0.33%) than that of panel ***B***.

Table S1. NGS qualified read numbers.

NGS Run	BC	Template	For Fig 2	Pol	Qualified reads
21H	60A	A	TMZ x1	d	26,896
21H	61A	B	TMZ x1	d	49,270
22B	1C	C	TMZ x1	d	11,484
22B	1D	D	TMZ x1	d	24,415
22B	1E	E	TMZ x1	d	20,486
22B	1F	F	TMZ x1	d	8,435
22B	1G	G	TMZ x1	d	11,747
24E	70a	A	TMZ x3	d	16594
24E	70b	B	TMZ x3	d	14767
24E	70c	C	TMZ x3	d	12858
24E	70d	D	TMZ x3	d	19080
24E	70e	E	TMZ x3	d	19995
24E	70f	F	TMZ x3	d	15417
24E	70g	G	TMZ x3	d	16527
23D	70a	A	TMZ x3 + hMGMT	d	4292
23D	70b	B	TMZ x3 + hMGMT	d	12532
23D	70c	C	TMZ x3 + hMGMT	d	5387
23D	70d	D	TMZ x3 + hMGMT	d	19676
23D	70e	E	TMZ x3 + hMGMT	d	11912
23D	70f	F	TMZ x3 + hMGMT	d	4547
23D	70g	G	TMZ x3 + hMGMT	d	8285
24E	60a	A	TMZ x3 (ssDNA)	d	6998
24E	60b	B	TMZ x3 (ssDNA)	d	5171
24E	60c	C	TMZ x3 (ssDNA)	d	4826
24E	60d	D	TMZ x3 (ssDNA)	d	9282
24E	60e	E	TMZ x3 (ssDNA)	d	9168
24E	60f	F	TMZ x3 (ssDNA)	d	7447
24E	60g	G	TMZ x3 (ssDNA)	d	8705

		Template	For Fig 3	Pol	Qualified reads
19A	30	O6-meG	defined damage	d	2100
19A	31	O6-meG	defined damage	e	1760
19A	32	O6-meG	defined damage	h	9840
19A	34	O6-meG	defined damage	z	5532

19A	35	O6-meG	defined damage	k	19474
19A	37	O6-meG	defined damage	d/h	4788
19A	38	O6-meG	defined damage	d/z	8908
19A	39	O6-meG	defined damage	d/k	16450

		Template	**For Fig 4**	Pol	Qualified reads
24E	72	A	-MGMT	d/z	20743
24E	72	B	-MGMT	d/z	20212
24E	72	C	-MGMT	d/z	18069
24E	72	D	-MGMT	d/z	24796
24E	72	E	-MGMT	d/z	25601
24E	72	F	-MGMT	d/z	21411
24E	72	G	-MGMT	d/z	25135

24E	73	A	-MGMT	d/k	19422
24E	73	B	-MGMT	d/k	17854
24E	73	C	-MGMT	d/k	14043
24E	73	D	-MGMT	d/k	19453
24E	73	E	-MGMT	d/k	21064
24E	73	F	-MGMT	d/k	17585
24E	73	G	-MGMT	d/k	17037

24E	71	A	-MGMT	d/h	10476
24E	71	B	-MGMT	d/h	9921
24E	71	C	-MGMT	d/h	7871
24E	71	D	-MGMT	d/h	12178
24E	71	E	-MGMT	d/h	12587
24E	71	F	-MGMT	d/h	9435
24E	71	G	-MGMT	d/h	10197

24E	12	A	+MGMT	d/z	14303
24E	12	B	+MGMT	d/z	24735
24E	12	C	+MGMT	d/z	42061
24E	12	D	+MGMT	d/z	116535
24E	12	E	+MGMT	d/z	116669
24E	12	F	+MGMT	d/z	76354
24E	12	G	+MGMT	d/z	123085

24E	16	A	+MGMT	d/k	10313

24E	16	B	+MGMT	d/k	17710
24E	16	C	+MGMT	d/k	28667
24E	16	D	+MGMT	d/k	78013
24E	16	E	+MGMT	d/k	81807
24E	16	F	+MGMT	d/k	52296
24E	16	G	+MGMT	d/k	87455

24E	72	A	+MGMT	d/h	13429
24E	72	B	+MGMT	d/h	22246
24E	72	C	+MGMT	d/h	41388
24E	72	D	+MGMT	d/h	114459
24E	72	E	+MGMT	d/h	123962
24E	72	F	+MGMT	d/h	78261
24E	72	G	+MGMT	d/h	122773

		Template	**For Fig. 5**	Pol	Qualified reads
25C	60	A	No damage	Z only	27,881
25C	60	B	No damage	Z only	19,448
25C	60	C	No damage	Z only	11,273
25C	60	D	No damage	Z only	16,550
25C	60	E	No damage	Z only	27,319
25C	60	F	No damage	Z only	17,712
25C	60	G	No damage	Z only	17202

25C	62	A	No damage	h-only	16,703
25C	62	B	No damage	h-only	11,679
25C	62	C	No damage	h-only	8,833
25C	62	D	No damage	h-only	13,954
25C	62	E	No damage	h-only	17,686
25C	62	F	No damage	h-only	11,249
25C	62	G	No damage	h-only	11105

25C	68	A	TMZx3	Z only	33,801
25C	68	B	TMZx3	Z only	35,372
25C	68	C	TMZx3	Z only	23,747
25C	68	D	TMZx3	Z only	35,215
25C	68	E	TMZx3	Z only	41,918
25C	68	F	TMZx3	Z only	30,666
25C	68	G	TMZx3	Z only	31689

25C	71	A	TMZx3	h-only	15842
25C	71	B	TMZx3	h-only	18,020
25C	71	C	TMZx3	h-only	12,774
25C	71	D	TMZx3	h-only	20,971
25C	71	E	TMZx3	h-only	20,792
25C	71	F	TMZx3	h-only	11,342
25C	71	G	TMZx3	h-only	14,425
